# Supplementary material for: Structural Basis of the Inhibition of L-Methionine γ-Lyase from Fusobacterium nucleatum
Source: Int J Mol Sci. 2023 Jan 13;24(2):1651. doi: 10.3390/ijms24021651 (PMC9865163; doi:10.3390/ijms24021651)
Supplement: Supplementary file 1 [file ijms-24-01651-s001.zip › ijms-2143543-supplementary.pdf]

Supplementary Data

Structural Basis of the Inhibition of L-methionine γ-lyase from *Fusobacterium nucleatum*

Tingting Bu [1,2†], Jing Lan [1,2†], Inseong Jo [3], Jie Zhang [1,2], Xue Bai [1,2], Shanru He [1,2], Xiaoling Jin [1], Lulu Wang [1,2,4], Yu Jin [1,2], Xiaoyu Jin [1,2], Liying Zhang [1,2], Hai-long Piao [4], Nam-Chul Ha [3], Chunshan Quan [1,2,*], Ki Hyun Nam [5,6,*], and Yongbin Xu [1,2,*]

[1]Department of Bioengineering, College of Life Science, Dalian Minzu University, Dalian 116600, Liaoning, China

[2]Key Laboratory of Biotechnology and Bioresources Utilization of Ministry of Education, College of Life Science, Dalian Minzu University, China

[3]Department of Agricultural Biotechnology, College of Agriculture and Life Sciences, Seoul National University, Gwanak-gu, Seoul 00826, Republic of Korea

[4]CAS Key Laboratory of Separation Science for Analytical Chemistry, Dalian Institute of Chemical Physics, Chinese Academy of Sciences, Dalian 116023, Liaoning, China

[5]Department of Life Science, Pohang University of Science and Technology, 35398 Pohang, Republic of Korea

[6]POSTECH Biotech Center, Pohang University of Science and Technology, 35398 Pohang, Republic of Korea

*For correspondence: Yongbin Xu, yongbinxu@dlnu.edu.cn; Ki Hyun Nam, structures@postech.ac.kr; Chunshan Quan, mikyeken@dlnu.edu.cn.

[†]Both authors contributed equally to this work.

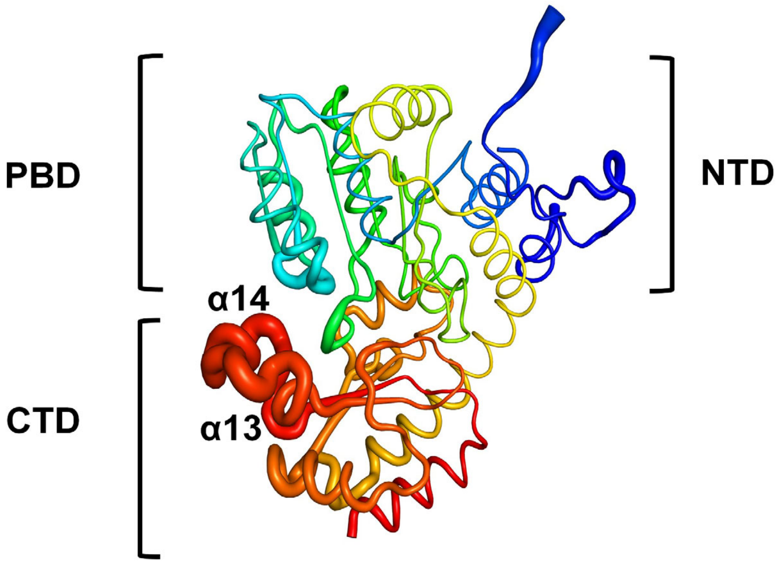

Figure S1. Analysis of the flexible region of Fn1419. Thermal parameter (B-factor) distribution in Fn1419. The Cα B-factors are depicted using colors ranging from dark blue (lowest B-factor) through to red (highest B-factor), with the radius of the ribbon increasing with the increase in the B-factor. The highest B-factor was observed in the region of the α-helix 13 (α13) and α-helix 14 (α14).

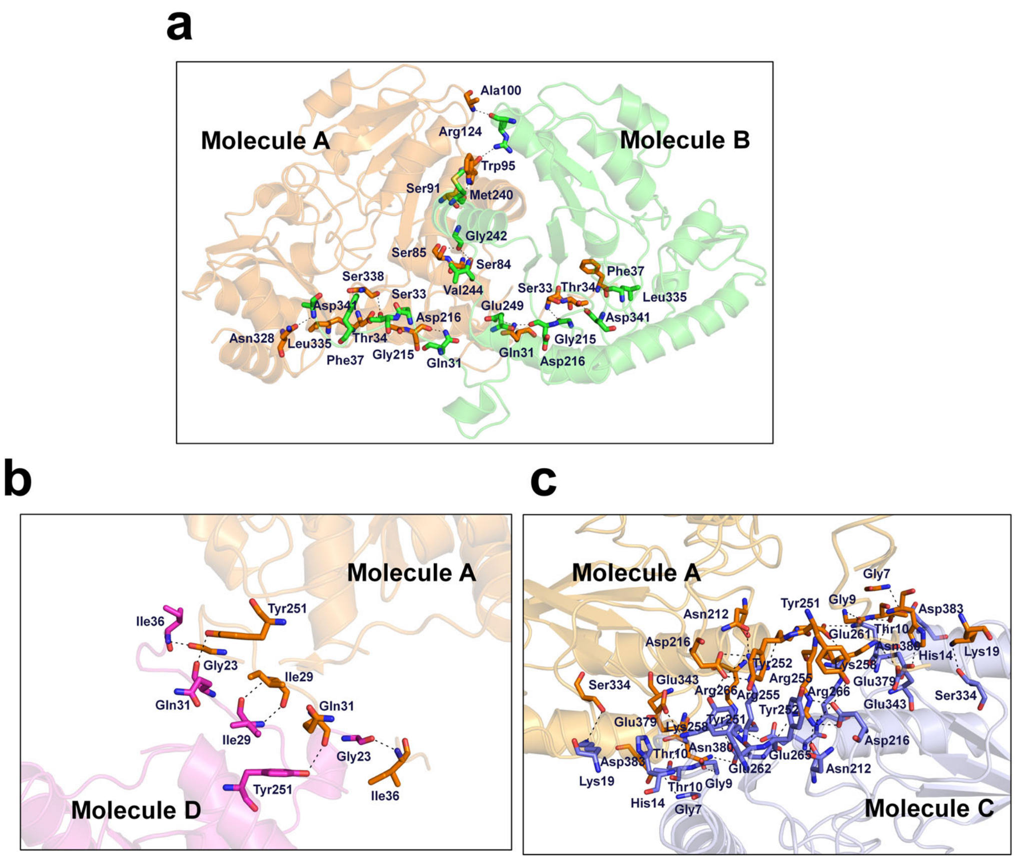

Figure S2. Analysis of Fn1419 dimer–dimer interactions. The dimer–dimer interfaces of Fn1419 (a) molecules A and B, (b) molecules A and D, and (c) molecules A and C are shown diagrammatically. The dimer interfaces with the different monomers are indicated using different colors. The residues involved in dimer–dimer interactions are shown as stick models and the hydrogen bonds as black broken lines.

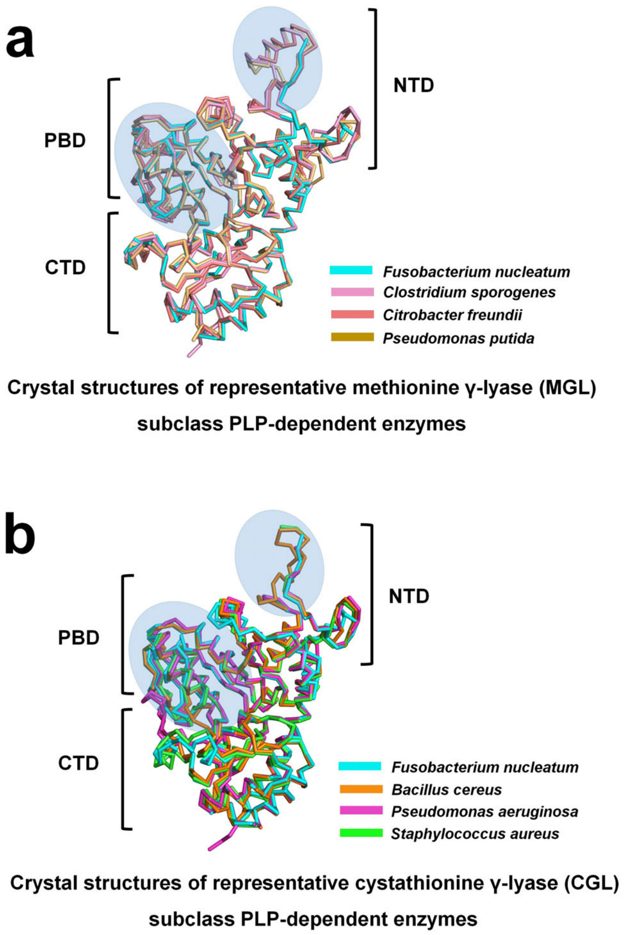

Figure S3. Superimposition of crystal structures of Fn1419 **homologous proteins.** (a) Crystal structures of representative L-methionine γ-lyase (MGL): *Clostridium sporogenes* (PDB ID: 5DX5), *Citrobacter freundii* (PDB ID: 6S0C), and *Pseudomonas putida* (PDB ID: 5X2W). (b) Crystal structures of representative cystathionine γ-lyase (CGL): *Bacillus cereus* (PDB ID: 7D7O), *P. aeruginosa* (PDB ID: 7BA4), and *Staphylococcus aureus* (PDB ID: 6KGZ). The flexible loops of the N-terminal domains and the PLP-binding domains are marked using different shades of blue.

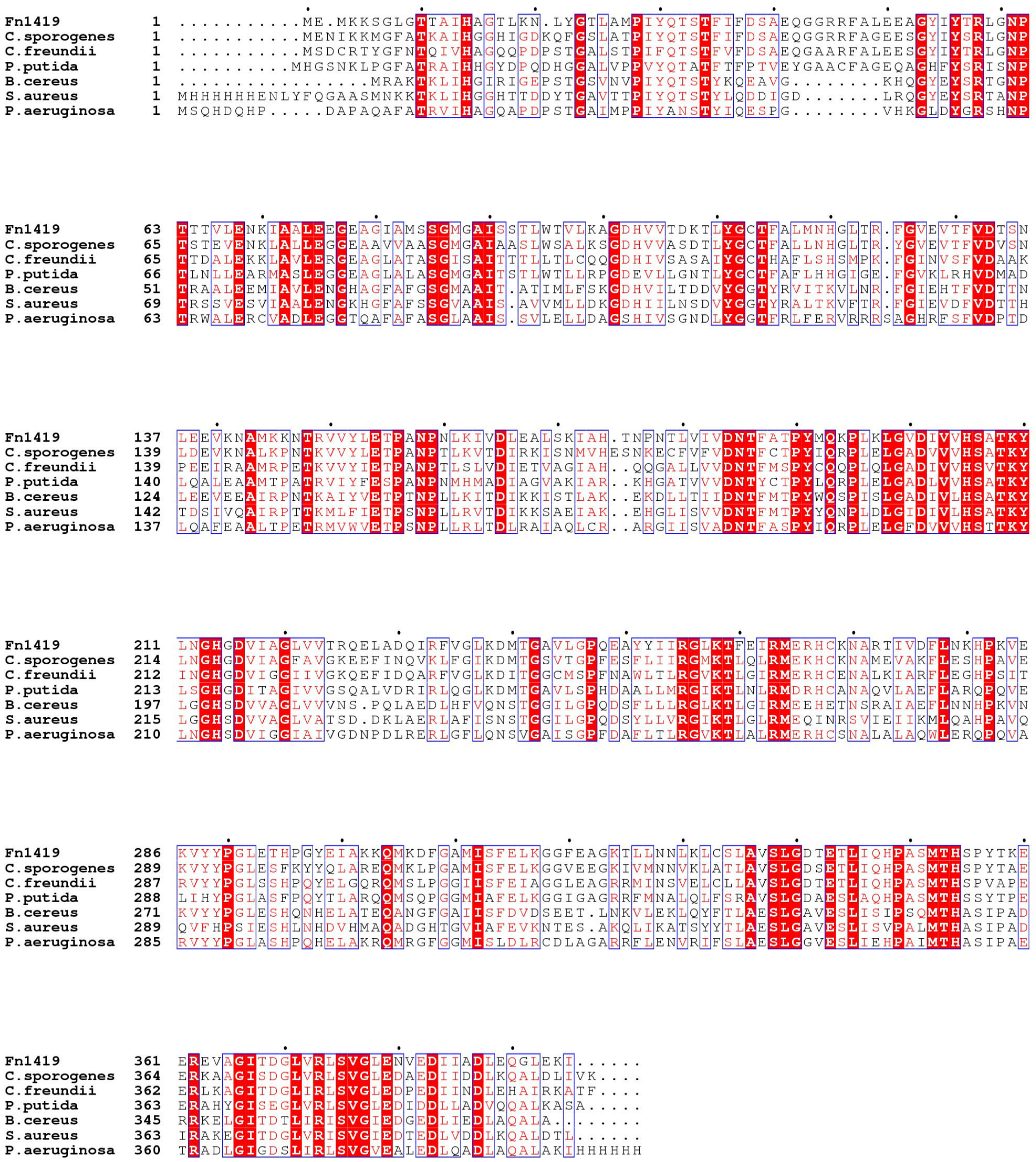

Figure S4. Sequence alignment of Fn1419 and homologous proteins. Sequence alignment of Fn1419 (Uniprot accession no. Q8RDT4), *Pseudomonas putida* MGL (P13254), *Citrobacter freundii* MGL (Q84AR1), *Clostridium sporogenes* MGL (A0A1J1CYV7), *Bacillus cereus* CGL (Q818A3), *Staphylococcus aureus* CGL (SAV0460), and *Pseudomonas aeruginosa* CGL (A0A2R3IX25).

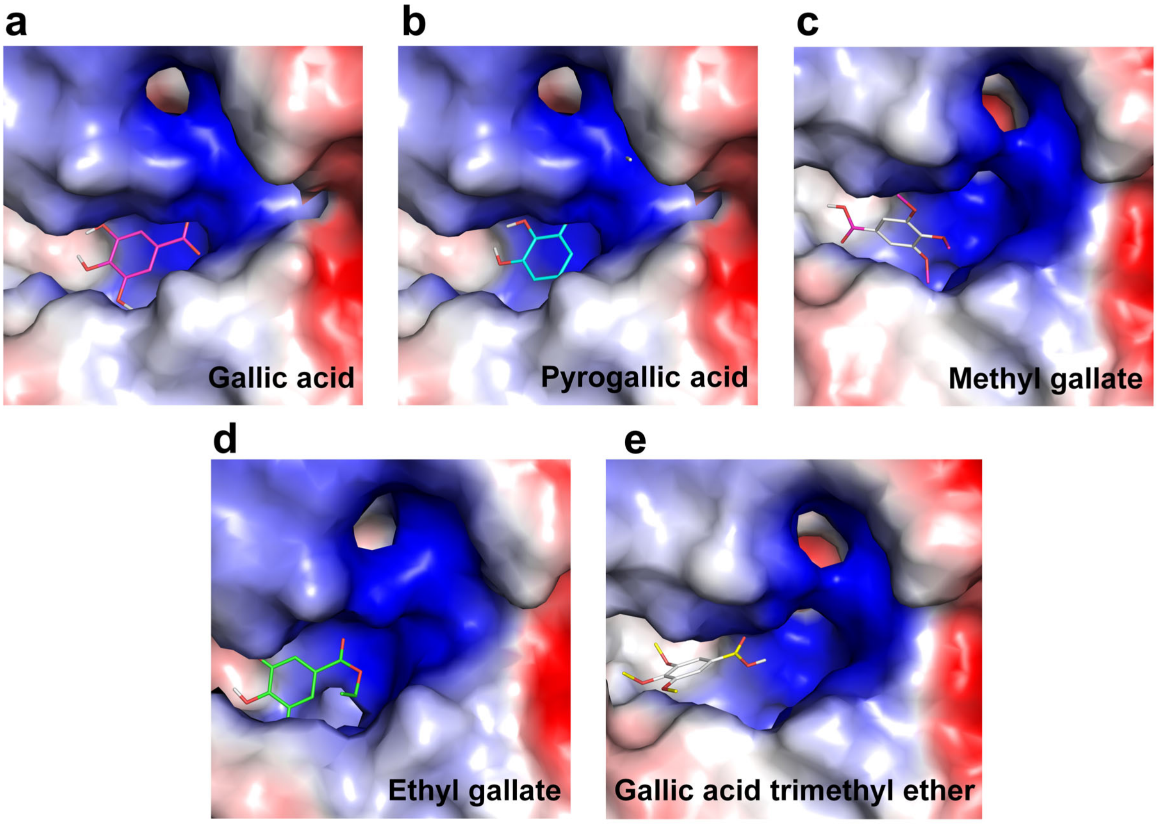

Figure S5. Electrostatic surface analysis of the Fn1419-binding pocket for natural product inhibitors. Pocket modelling for gallic acid (a), pyrogallic acid (b), methyl gallate (c), ethyl gallate (d), and gallic acid trimethyl ether (e) is shown.

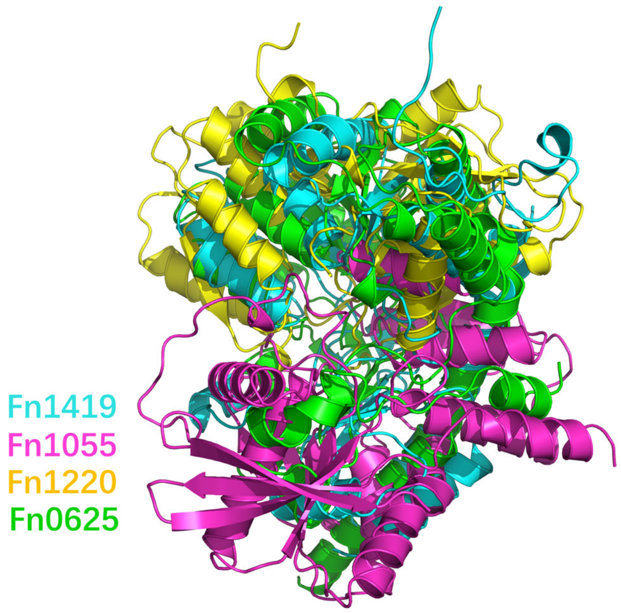

Figure S6. Superimposition of structures of PLP-dependent H_2S-producing enzymes from *Fusobacterium nucleatum*. Superimposition of Fn1419 (PDB ID: 7BQW), Fn1055 (5B53), Fn1220 (5XEO), and Fn0625 (AlphaFold prediction) is presented.

Table S1. Hydrogen bonds and salt bridges of dimer-interface interactions of Fn1419

A. Hydrogen bonds between molecule A and molecule B

molecule A	molecule B	length (Å)
S338 OG	S33 O	3.71
D216 N	S33 OG	3.67
A100 N	R124 O	2.96
T32 OG1	G215 O	3.73
S33 N	G215 O	3.02
T34 N	G215 O	3.70
T32 N	D216 O	3.84
Q31 NE2	D216 O	2.64
W95 NE1	M240 O	2.93
S91 OG	T241 O	3.44
S85 OG	G242 O	2.38
S85 N	G242 O	3.78
S84 OG	G242 O	3.27
F37 N	L335 O	2.91
T34 OG1	D341 OD2	2.33
D216 O	Q31 NE2	2.56
G215 O	T32 OG1	3.62
G215 O	S33 N	2.81
D341 OD2	T34 OG1	2.79
L335 O	F37 N	3.04
N328 OD1	A40 N	3.25
W95 O	R124 NH1	3.17
S33 OG	D216 N	3.64
C113 SG	K238 NZ	3.45
S84 OG	V244 N	3.28

B. Hydrogen bonds between molecule A and molecule D

molecule A	molecule D	length (Å)
L25 N	T34 O	3.90
L29 N	I29 O	3.04
L36 N	G23 O	2.82
Y251 OH	Q31 O	3.58
G23 O	I36 N	2.48
I29 O	I29 N	2.85
Q31 O	Y251 OH	3.31
T34 O	L25 N	3.90

C. Hydrogen bonds and salt bridges between molecule A and molecule C

molecule A	molecule C	length (Å)
N380 N	T10 OG1	3.12
R255 NH2	N212 OD1	3.03
R255 NH2	D216 OD1	2.46
R255 NE	D216 OD2	2.54
Y251 OH	D216 OD2	2.66
R255 NH1	Y252 O	3.11
Q248 NE2	Y252 OH	2.84
N380 ND2	E261 OE1	3.02
R266 NH2	E261 OE2	2.87
R266 NH1	E265 OE2	2.94
K19 NZ	S334 OG	2.92
K258 NZ	E343 OE1	3.40
H14 NE2	E379 OE2	2.77
L8 N	N380 OD1	3.12
T10 OG1	N380 OD1	3.57
G9 N	N380 OD1	2.80
T10 N	N380 OD1	3.30
G7 N	D383 OD2	2.91
T10 OG1	D383 OD2	2.67
T10 OG1	N380 N	3.04
N212 OD1	R255 NH2	2.79
D216 OD1	R255 NH2	2.72
D216 OD2	Y251 OH	2.71
D216 OD2	R255 NE	2.61
Y252 O	R255 NH1	3.00
Y252 O	R255 NH2	3.17
Y252 OH	Q248 NE2	3.25
E261 OE1	N380 ND2	3.26
E261 OE2	R266 NH1	3.09
E265 OE1	R266 NH2	3.73
S334 OG	K19 NZ	3.56
E343 OE1	K258 NZ	2.85
E379 OE2	H14 NE2	2.70
N380 OD1	T10 N	3.60
N380 OD1	L8 N	2.95
N380 OD1	G9 N	2.83
D383 OD2	G7 N	2.92
D383 OD2	T10 OG1	2.82
R255 NE	D216 OD1	3.62
R255 NH2	D216 OD1	2.46

R255 NE	D216 OD2	2.54
R255 NH2	D216 OD2	2.94
R266 NH1	E261 OE1	3.58
R266 NH1	E261 OE2	2.83
R266 NH2	E261 OE2	2.87
R266 NH1	E265 OE2	2.94
K258 NZ	E343 OE1	3.40
H14 NE2	E379 OE2	2.77
D216 OD1	R255 NE	3.71
D216 OD1	R255 NH2	2.72
D216 OD2	R255 NE	2.61
D216 OD2	R255 NH2	3.22
E261 OE2	R266 NH2	3.52
E261 OE2	R266 NH1	3.09
E265 OE1	R266 NH2	3.73
E343 OE1	K258 NZ	2.85
E379 OE2	H14 NE2	2.70

Table S2. Oligonucleotide Sequence of Primers.

**Primers**	**Sequence (5'-3')**
**F-Fn1419-WT**	GGGCCATGGATCACCATCACCATCACCATATGGAAAT[illegible] AAAAATCTGGTTTAG
**R-Fn1419-WT**	GGGCTCGAGTTAAATTTTTTCTAGTCCTTGTTCTAAAT
**F-Fn1419-Y111A**	GTTGTTACAGATAAAACTTTAGCTGGTTGTACTTTTGC[illegible] TGATGAATCAT
**R-Fn1419-Y111A**	ATTCATCAAAGCAAAAGTACAACCAGCTAAAGTTTTA[illegible] TGTAACAAC
**F-Fn1419-G112A**	GTTACAGATAAAACTTTATATGCTTGTACTTTTGCTTT[illegible] TG
**R-Fn1419-G112A**	CATCAAAGCAAAAGTACAAGCATATAAAGTTTTATCT[illegible] AAC
**F-Fn1419-P159A**	CTTGAAACTCCTGCCAATGCAAATTTAAAAATAG
**R-Fn1419-P159A**	CTATTTTTAAATTTGCATTGGCAGGAGTTTCAAG
**F-Fn1419-V337A**	TTAAAACTTTGTTCATTAGCAGCTTCATTAGGAGATAC[illegible] AAACTC
**R-Fn1419-V337A**	GAGTTTCAGTATCTCCTAATGAAGCTGCTAATGAACA[illegible] GTTTTAA
**F-Fn1419-S338A**	CTTTGTTCATTAGCAGTTGCATTAGGAGATACTGAAA[illegible] CTTATTCAACAC
**R-Fn1419-S338A**	GTGTTGAATAAGAGTTTCAGTATCTCCTAATGCAACT[illegible] TAATGAACAAAG
**F-Fn1419-R373A**	GGAATCACTGATGGTTTAGTTGCTTTATCAGTTGGAC[illegible] GAAAAT
**R-Fn1419-R373A**	ATTTTCAAGTCCAACTGATAAAGCAACTAAACCATCA[illegible] GATTCC

The *Nco*I and *Xho*I restriction sites are underlined. The mutant sites are marked red color with double-underlined.
